# Association between sense of coherence and health-related quality of life among primary care patients with chronic musculoskeletal pain

**DOI:** 10.1186/1477-7525-11-216

**Published:** 2013-12-26

**Authors:** Neale R Chumbler, Kurt Kroenke, Samantha Outcalt, Matthew J Bair, Erin Krebs, Jingwei Wu, Zhangsheng Yu

**Affiliations:** 1 Department of Health Policy and Management, University of Georgia, Clayton Street, Office 305, Athens, GA 30602, USA; 2 VA HSR&D Center for Health Information and Communication, Roudebush VA Medical Center, Indianapolis, IN, USA; 3 Department of Medicine, Indiana University School of Medicine, Indianapolis, IN, USA; 4 Regenstrief Institute, Inc., Indianapolis, IN, USA; 5 VA HSR&D Center for Chronic Disease Outcomes Research, Minneapolis VA Health Care System, Minneapolis, MN, USA; 6 University of Minnesota Medical School, Minneapolis, MN, USA; 7 Department of Biostatistics, Indiana University School of Medicine, Indianapolis, IN, USA

## Abstract

**Background:**

Sense of Coherence (SOC) is a measure of an individual’s capacity to use various coping mechanisms and resources when faced with a stressor. Chronic pain is one of the most prevalent and disabling conditions in clinical practice. This study examines the extent to which a strong SOC is associated with less pain and better health related quality of life (HRQoL) among patients with chronic pain.

**Methods:**

We analyzed data from the Stepped Care to Optimize Pain care Effectiveness (SCOPE) trial which enrolled 250 patients with persistent (3 months or longer) musculoskeletal pain who were receiving care in an United States Department of Veterans Affairs (VA) primary care clinic. The abbreviated three-item SOC scale was used to measure personal coping capability. Participants were categorized into Strong SOC (score 0–1) and Weak SOC (score 2–6). The Brief Pain Inventory (BPI) was used to assess the severity and disability associated with pain. Additionally, pain self-efficacy (ASES) and catastrophizing (CSQ) were assessed. HRQoL was assessed with the 36-item Short-Form Health Survey (SF-36) social functioning, vitality, and general health subscales. Multiple linear regression models were performed to examine whether SOC was independently associated with pain-specific and HRQoL outcomes, after adjusting for sociodemographic and socioeconomic characteristics, medical comorbidities and major depression.

**Results:**

Of the 250 study patients, 61% had a strong SOC whereas 39% had a weak SOC. Multivariable linear regression analysis showed that a strong SOC was significantly associated with better general health, vitality, social functioning and pain self-efficacy as well as less pain catastrophizing. These significant findings were partially attenuated, but remained statistically significant, after controlling for major depression. SOC was not significantly associated with pain severity or pain disability.

**Conclusions:**

A strong SOC is associated with better HRQoL and self-efficacy as well as less catastrophizing in patients with chronic pain. SOC may be an important coping mechanism (strategy) for patients with chronic musculoskeletal pain.

**Trial registration:**

Clinicaltrials.gov Identifier: NCT00926588.

## Introduction

Musculoskeletal pain is the most common, frequently occurring, disabling and costly of all pain complaints in the primary care setting [1]. Nearly two-thirds of pain-related visits are the result of musculoskeletal pain, representing around 70 million outpatient visits in the U.S. each year [2]. In fact, back pain and joint pain alone lead to approximately 200 million lost work days per year [3,4]. Although pain medications are the second most common prescribed class of drugs in the US primary care setting, analgesics fail to provide adequate relief for many patients [5,6]. Thus, an important aspect of managing chronic pain includes strategies for enhancing coping strategies, reducing pain-related impairment, and improving health-related quality of life (HRQoL) and functional status [3,6].

Central to understanding HRQoL and functional status is the fact that certain social factors may maintain health and prevent adverse health consequences. Antonovsky [7] categorized this as the *salutogenic orientation*. This orientation views the presence of stressors as not an abnormality but rather a pervasive condition for individuals [8]. Antonovsky proposed that Sense of Coherence (SOC) is a key variable in maintaining health [7], postulating SOC as a dispositional orientation (i.e., orientation to life) that can help avert breakdown in stressful conditions through the use of appropriate coping responses [9]. In this sense, SOC is a global measure that indicates the availability of, and willingness to use, adaptive coping resources [8]. SOC has three components: 1) comprehensibility (belief that what happens in life is rational, predictable, and understandable); 2) manageability (belief that resources are available to help resolve difficulties as they arise); and 3) meaningfulness (adversity seen as a challenge and such a difficulty is worthy of engagement). Thus, individuals with a strong SOC, relative to those with a weak SOC, will be more likely to consider stressors as predictable, have confidence in their ability to triumph over stressors, and consider it important to rise to the trials they face [10]. As with all personality aspects, the SOC has its origin in both patterned sociocultural and psychological circumstances, as well as unique events [8]. The SOC is one key adaptive coping response that may be important in the future development of interventions for individuals with chronic pain. In this regard, SOC is more than just a coping approach, but a factor that also leads an individual to engage in healthy behaviors [10].

According to Antonovsky [9], individuals with a stronger SOC, despite extremely challenging circumstances, can effectively handle stress and protect their health. Thus, individuals with musculoskeletal pain who possess a strong SOC may be better able to respond to stressors by employing adaptive coping resources and, consequently, have better HRQoL [11]. Theoretically, a strong SOC could protect individuals with impairments, such as pain conditions, from dissatisfaction with life by supporting, if necessary, reorientation towards new goals that improve life satisfaction.

Despite the potential benefits of SOC, there have been few studies examining the effects of SOC in patients with chronic pain, including its association with HRQoL and functional status. In an observational study of Norwegian patients with various musculoskeletal pain conditions referred from primary care physicians to a Physical Medicine and Rehabilitation unit, Anke et al. [12] found that strong SOC was associated with lower pain intensity and higher levels of life satisfaction. Conversely, these same authors argued that SOC may be negatively affected by persistently painful conditions, thereby reducing an individuals’ coping ability (i.e., SOC).

The aim of the present study was to determine if SOC is independently associated with pain and HRQoL outcomes in patients with chronic pain. More specifically, we postulated that a strong SOC is positively associated with better pain-specific as well as general HRQoL outcomes. Since some researchers have challenged whether SOC is truly a distinct salutogenic construct, but rather an inverse measure of persistent depressive symptoms [13], we also controlled for the presence of major depression. In contrast to previous studies that have primarily assessed measures of depressive and other psychological symptoms [14], we used a criterion-based diagnosis of major depression. A strong body of literature has found that a strong SOC is correlated with fewer symptoms of perceived depression and HRQoL [15]. We also controlled for self-efficacy, pain catastrophizing and demographic variables. Past research found that SOC correlates positively with self-efficacy [16]. Even though no previous studies have examined the association between SOC and pain catastrophizing, Benz et al. [17] found that self-report pain to be inversely correlated with SOC. With reference to demographic factors, some studies have found that strong SOC was associated with older age [18], being male [18], greater educational attainment [19], being married [20], and being employed [20]. We also capitalized on the longitudinal data available in the study to examine if SOC changed over 12 months.

## Methods

### Study design and participation

This paper draws upon data from a randomized clinical effectiveness trial referred to as the *Stepped Care to Optimize Pain care Effectiveness* (SCOPE) study, which is described in detail elsewhere. This RCT was carried out in accordance with the Code of Ethics of the World Medical Association (Declaration of Helsinki) [3]. This study was reviewed and approved by the Indiana University Institutional Review Board and the VA Research Review Committee. In brief, SCOPE enrolled primary care patients with clinically significant musculoskeletal pain who, after providing informed consent and completing a baseline interview, were randomized to either a stepped care optimized analgesic arm or a usual care control arm. Study participants were veterans 18 to 65 years of age who received care from one of five primary care clinics at a large United States Department of Veterans Affairs (VA) Medical Center in the Midwest. To be eligible, patients had to have pain which was: (1) *musculoskeletal* defined as regional (e.g., low back or joints) or more generalized (e.g., fibromyalgia); (2) at least *moderate to severe* in intensity (i.e., defined as a Brief Pain Inventory severity score ≥ 5 of either the patient’s average or worst pain in the past week [21]; and (3) *persistent* (i.e., ≥ 3 months in duration despite trying at least one analgesic medication). Individuals were excluded if they (a) had a pending pain-related VA or Social Security disability claim; (b) did not speak English; (c) had moderately severe cognitive impairment; (d) had schizophrenia, bipolar disorder, or other psychosis; (e) were actively suicidal; (f) had current illicit drug use; or (g) had an anticipated life expectancy of less than 12 months.

Physicians who worked in the five primary care clinics were informed of the study and asked if their patients could be contacted for potential participation in the study. For physicians who agreed, their patients who had electronic medical record evidence of a primary care visit in the past 12 months and an International Classification of Diseases (ICD-9) diagnosis of musculoskeletal pain were sent a letter describing details of the study, along with a form that could be returned expressing potential interest in study participation. A flow chart detailing screening, eligibility determination and enrollment has been previously published [3]. More simply put, over a two-year period, letters were mailed to 940 patients, of whom 311 were contacted by phone (most of whom had returned the form expressing their potential interest in the study). Sixty-one patients were not enrolled (29 whose pain did not meet the pain severity threshold; 22 who were eligible but not interested in participating; and 10 who did not complete an eligibility interview). Thus, 250 patients were eligible and enrolled in the study. Randomization resulted in comparable groups on all measured variables. After providing written informed consent, the study participants completed a baseline interview and then were randomized to either the intervention or usual care arm.

### Study measures

Three measures were used to assess pain-specific outcomes. The *Brief Pain Inventory* (BPI) consists of two scales that measure pain intensity and pain-related functional impairment (physical and emotional) [22-24]. The BPI severity scale evaluates the intensity of pain with 4 items (current, worst, least, and average pain in the past week) on scales from 0 (“no pain”) to 10 (“pain as bad as you can imagine”). The BPI interference scale measures pain-related functional impairment across 7 unique domains (mood, physical activity, walking ability, normal work, relations with other people, sleep, and enjoyment of life) rated from 0 (“does not interfere”) to 10 (“interferes completely”). Each BPI subscale score is the mean of its component items and can range from 0 to 10. The 6-item *Arthritis Self-Efficacy Scale (ASES)* measures one’s beliefs regarding the ability to manage pain symptoms [25]. Each of the 6 items is scored on an interval scale with scores varying from 1 (“very uncertain) to 10 (“very certain”). An overall ASES score is calculated by the mean of the 6 items.

The 6-item catastrophizing subscale of the Coping Strategies Questionnaire (CSQ) was employed to measure pain catastrophizing cognitions (e.g., “I worry all the time about whether it will end”) [26,27]. Study participants evaluated the extent to which they engage in that activity or thought when experiencing pain, on a scale from 0–6 (“never do that” to “always do that”). The subscale score is calculated as the total of all 6 items, and higher scores indicate more frequent pain catastrophizing.

HRQoL was assessed with three subscales of the 36-item Short-Form Health Survey (SF-36): general health, vitality, and social functioning [28]. Each SF score ranges from 0 to 100, with higher scores indicating better health. HRQoL was also measured with the SF-12 (which provides both Physical Component Summary and Mental Component Summary scores) [29].

We used an abbreviated 3-item version of the SOC scale [30] which assesses each of the component constructs by single questions: 1) “Do you usually feel that the things that happen to you in your daily life are hard to understand?” (*comprehensibility*); 2) “Do you usually see a solution to problems and difficulties that other people find hopeless?” (*management*); and 3) “Do you usually feel that your daily life is a source of personal satisfaction?” (*meaningfulness*). Study participant responses were “yes, usually” (scored 0); “yes, sometimes” (scored 1); and “no” (scored 2). Following reverse scoring for comprehensibility, all items were summed to provide a total SOC scale score in the range of 0–6; a higher score represents a weaker SOC (Cronbach’s α = .58). Following previous research [31], SOC was recoded as a binary variable, where a score of 0 or 1 represents “strong SOC” and a score of 2–6 “weak SOC”.

The following demographic variables were collected by self-report at baseline and used in the present analyses as covariates: 1) age (in years); 2) sex; 3) race (white vs. black/other); and 4) medical comorbidity index (a checklist of eight common categories of medical conditions that include heart disease, pulmonary disease, diabetes mellitus, hypertension, neurologic conditions, arthritis, liver disease, and renal disease) [32]. The socioeconomic disadvantage index is a measure that encompasses three self-report items with reference to an individual’s educational attainment, employment status, and income (“comfortable”, “just enough to make ends meet”, and “not enough to make ends meet”). This index is created by assigning 1 point each for low education (high school degree or less), unemployment, and low income (“just enough or not enough to ends meet”). Higher scores on this 0 to 3 scale represent worse socioeconomic conditions [33]. The Patient Health Questionnaire (PHQ-9) categorical scoring algorithm was used to determine the presence of probable major depressive disorder [34]. The PHQ-9 algorithm has been shown to conform well to criterion-standard diagnoses of major depression [33].

### Statistical analysis

SOC was defined as a binary variable (strong vs. weak SOC) and was analyzed as the main predictor in all analyses. First, univariate associations with pain-specific and HRQoL domains were evaluated by comparing these domains between strong and weak SOC patients. Next, multiple linear regression models were fitted to test the independent association of SOC with each of the 7 pain-specific and HRQoL domains as dependent variables. For each domain, two models were fitted. *Step 1* adjusted for patient age, sex, race, socioeconomic disadvantage index, and medical comorbidity. *Step 2* adjusted for the same variables as in Step 1 plus the presence or absence of major depression. All analyses were conducted using SAS version 9.3 (SAS Institute, Cary, North Carolina). Analyses of the measures were performed based on an intention-to-treat strategy. Group differences in the outcome total scores were estimated using mixed effects model repeated measures analysis. Missing data was minimal because the assessments were interviewer-administered. Also, follow-up interviews were completed in 98% of participants at 3 months and 95% at 12 months.

## Results

Of the 250 study participants, 61% (n = 152) had a strong SOC (score, 0 or 1) and 39% (n = 98) had a weak SOC (score, 2–6). The proportion of participants with a score of 0, 1, 2, 3, 4, and 5–6 was 34.4%, 26.4%, 19.2%, 9.2%, 6.4%, and 4.4%, respectively. The study participants had a mean age of 55.1 years (SD = 8.5; age range, 28–66 years); 82.8% were men, 76.8% were white. The mean BPI severity score was 5.1 (SD = 1.7), and the mean BPI interference score was 5.3 (SD = 2.2). The mean number of comorbid medical diseases was 2.2 (SD = 1.3), and 24% of participants had major depression. The duration of chronic pain was 3–12 months in 2.0% (n = 5) patients, 1–5 years in 26.4% (n = 66), 6–10 years in 19.2% (n = 48), and more than 10 years in 52.4% (n = 131).

Table 1 summarizes the univariate associations of SOC with pain and HQRoL. A strong SOC was highly associated with lower pain intensity and impairment, better pain self-efficacy and less pain catastrophizing. A strong SOC was also highly associated (P < .001) with better HRQoL on all three SF-36 domains--- general health, vitality, and social functioning--- and in the SF-12 MCS.

**Table 1:** Comparison of chronic pain patients with strong and weak sense of coherence (SOC)*

**Patient characteristic**	**Strong SOC**	**Weak SOC**	**P value**
	**(n = 152)**	**(n = 98)**	
Age in years, mean (SD)	55.4 (8.4)	54.9 (8.6)	.67
Male, n (%)	125 (82.2)	82 (83.7)	.77
White, n, (%)	118 (77.6)	74 (75.5)	.77
Education > high school, n (%)	123 (80.9)	62 (63.3)	.002
Married, n (%)	106 (69.7)	79 (80.6)	.056
Education > high school, n (%)	123 (80.9)	62 (63.3)	.002
Employed, n (%)	105 (69.1)	55 (56.1)	.019
Income adequate by self-report, n (%)	143 (94.1)	82 (83.7)	<.0001
Socioeconomic index, mean (SD)	0.7 (0.7)	1.3 (1.0)	<.0001
Major depression, n (%)	17 (11.2)	43 (43.9)	<.0001
Comorbid medical diseases	1.9 (1.3)	2.3 (1.3)	.015
Pain-specific scores, mean (SD)			
BPI severity [0–**10**]	5.0 (1.8)	5.3 (1.5)	.151
BPI interference [0–**10**]	4.9 (2.2)	5.9 (2.2)	.0004
ASES pain self-efficacy [1-10]	6.9 (1.9)	5.3 (2.2)	<.0001
CSQ pain catastrophizing [0–**36**]	8.6 (6.5)	15.3 (9.2)	<.0001
Health related quality of life scores, mean (SD)^**†**^			
SF-36 general health [**0**–100]	58.6 (26.4)	39.9 (29.4)	<.0001
SF-36 vitality [**0**–100]	47.6 (21.0)	29.7 (20.7)	<.0001
SF-36 social functioning [**0**–100]	75.4 (22.8)	53.3 (28.1)	<.0001
SF-12 physical component [norm = 50]	35.9 (9.3)	34.8 (8.9)	<.343
SF-12 mental component [norm = 50]	53.4 (8.9)	40.3 (12.6)	<.0001

*Numbers in brackets represent minimum to maximum scores on each scale, with worst scorerepresented by the bolded number.

^**†**^Strong SOC = score of 0 to1. Weak SOC = score of 2 to 6.

BPI = *Brief Pain Inventory;* ASES = *Arthritis Self-Efficacy Scale;* CSQ = *Catastrophizing Strategies Questionnaire;* SF-36 = *the 36-item Short-Form Health Survey*; *SF-12 = the 12-item Short-Form Health Survey.*

Patients with a strong SOC were much less likely to have major depression, had fewer comorbid medical conditions, and had better educational, employment and income status. No association was observed between SOC and patient age, gender, or race.

Table 2 summarizes results from the multiple linear regression models. A strong SOC was associated with a 1.25 point increase in the ASES pain self-efficacy score (p < .0001). After adjusting for the presence of major depression (step 2), a strong SOC was associated with a somewhat smaller but still significant 0.70 increase in ASES score (p = .008). A strong SOC was also associated with a 5.36 point decrease in the CSQ pain catastrophizing score (p < .0001), which was attenuated to a 2.95 point decrease (p = .003) after adjusting for the presence of major depression. A strong SOC was associated with a 0.58 point decrease in the BPI interference subscale in step 1, but the association was no longer significant after adjusting for major depression. SOC was not associated with BPI measures of pain intensity or pain-related functional impairment in either the step 1 or step 2 models.

**Table 2:** Sense of coherence and major depression as predictors of pain specific outcomes and health-related quality of life in patients with chronic musculoskeletal pain

**Outcome (pain or HRQoL)**	**Adjusted (step 1)**^**A**^				**Adjusted (step 2)**^**B**^				**Adjusted (step 2)**^**B**^			
	***Strong SOC as the predictor***				***Strong SOC as the predictor***				***Absence of major depression as the predictor***			
	**Beta**	**SE**	**T-value**	**P**	**Beta**	**SE**	**T-value**	**P**	**Beta**	**SE**	**T-value**	**P**
BPI severity	−.064	.233	−0.27	.784	.178	.241	0.74	.461	-.880	.271	−3.25	.001
BPI interference	−.576	.292	−1.97	.050	.018	.282	.06	.949	−2.16	.317	−6.82	<.0001
ASES pain self-efficacy	1.245	.272	4.58	<.0001	.702	.263	2.67	.008	1.98	.296	6.68	<.0001
CSQ pain catastrophizing	−5.36	1.035	−5.18	<.0001	−2.95	0.968	−3.04	.003	−8.79	1.09	−8.07	<.0001
SF-36 general health	11.059	3.408	3.24	.001	7.503	3.514	2.14	.034	12.943	3.954	3.27	.001
SF-36 vitality	14.784	2.673	5.53	<.0001	10.683	2.685	3.98	<.0001	14.923	3.020	4.94	<.0001
SF-36 social functioning	20.589	3.415	6.03	<.0001	12.924	3.226	4.01	<.0001	27.897	3.629	7.69	<.0001
SF-12 mental component	12.083	1.433	8.38	<.0001	8.280	1.298	6.38	<.0001	13.839	1.461	9.48	<.0001

^A^Step 1: Adjusted for age, sex, race, sociodemographic disadvantage index, medical comorbidity.

^B^Step 2: Adjusted for variables in step 1, plus major depression.

Note: SOC = *Sense of Coherence;* BPI = *Brief Pain Inventory;* ASES = *Arthritis Self-Efficacy Scale;* CSQ = *Catastrophizing Strategies Questionnaire;* SF-36 = *the 36-item Short-Form Health Survey; SF-12* =.

A strong SOC was associated with relatively large increases in the scores on all three SF-36 HRQoL domains and in the SF-12 mental MCS; including an 11.1 point increase in general health, a 14.8 point increase in vitality, a 20.6 point increase in social functioning, and a 12.1 point increase in mental health. These increases remained moderate in magnitude (7.5, 10.7, 12.9, and 8.3, respectively) and statistically significant, even after adjusting for the presence of major depression.

The last column in Table 2 also shows the strength of association of major depression with pain and HRQoL outcomes. Note that the “absence of major depression” had a strong and independent association with better pain-specific as well HRQoL outcomes in all models. Inspection of the T-values in the step 2 models highlights the relative strengths of association of SOC and major depression with pain and HRQoL outcomes. The strength of association of a *strong SOC* with outcomes was closest to that of *absence of major depression* for vitality (ratio of t-scores = 3.98/4.94 or 81%), followed by SF-12 MCS (67%), general health (65%), social functioning (52%), and pain self-efficacy (40%).

Longitudinal analyses indicated that SOC did not change over the 12 months of the trial. For the intervention group, the mean SOC values essentially remained the same at baseline, 3-months and at 12-months (1.43, 1.37, and 1.47, respectively). Similarly, the mean values changes very little in the control group at baseline, 3-months, and at 12-months (1.39, 1.50, 1.42, respectively). The differences between these groups were not significant. The strong associations of SOC with pain and HRQoL outcomes present at baseline persisted at 3 and 12 months. Moreover, the HRQoL outcomes did not significantly change over the 12 months (results not shown).

## Discussion

Our study has several important findings. First, a strong SOC is associated with better pain-specific outcomes, especially for constructs relevant to coping such as better pain self-efficacy and less pain catastrophizing. Second, a strong SOC is also associated with better HRQoL, specifically self-reported general health, vitality, and social functioning. Third, the association of SOC remained significant for most outcomes (except pain intensity and impairment) even after controlling for important covariates, including the presence of major depression. Indeed, the strength of association of SOC with pain coping and HRQoL outcomes was 40-80% that of major depression. Fourth, SOC did not change over the 12 months of the trial, indicating that SOC was a stable trait in our study population.

Our findings are consistent with previous studies that have found an association between SOC and HRQoL. A systematic review published in 2007 found that a stronger SOC was associated with better HRQoL [35]. Our results in chronic pain patients complement findings in other medical populations confirming the association between SOC and HRQoL as measured by the SF-36, including coronary heart disease patients [36], patients following a myocardial infarction [37], nursing home residents [38], family caregivers of older adults living in the community [11], and middle-aged persons from the general population [39].

Similar to our study, a cross-sectional study of 232 participants recruited from a “Neck and Back” unit in Norway found that stronger SOC was associated with greater pain self-efficacy [12]. Prior studies have found mixed results for associations of SOC with pain intensity and functional impairment. Our findings are in line with those of Benz et al. [17], who found that SOC was unrelated to pain severity in a prospective cohort study of 355 patients with hip and knee osteoarthritis. In contrast, at least two studies have found associations of SOC with pain severity. A small sample of 73 patients who received laparoscopic cholecystectomy found that a strong SOC was significant, but weak predictor of less intense postoperative pain at one week [40]. Likewise, in another small study assessing the features of fibromyalgia syndrome in 40 women with non-metastatic breast cancer, SOC was inversely correlated with BPI severity and BPI interference [41]; however, this study did not adjust for sociodemographic or clinical variables (including depression).

Although low SOC does not inherently imply a depressive mood, it has been associated with depression in prior research [42]. In contrast to most previous studies, we were able to control for a clinical diagnosis of major depression, rather than simply a continuous measure of sub-threshold depressive symptoms. We found that SOC remained statistically significant for all outcomes (except for 2 pain outcomes), even after controlling for major depression. More specifically, after we adjusted for major depression, the T-value was attenuated, but remained statistically significant for five of the seven outcomes.

Because of the strong association between SOC and depression, some researchers have even questioned the extent to which they are conceptually distinct from one another [14,43]. For instance, in a recent national Finnish-based survey study, higher SOC scores were related to a lower risk of cardiovascular disease and mortality, but this association disappeared after adjustment for depressive symptoms [44]. One partial explanation for the independent effects of SOC found in our study may be that the measure we used – the SOC-3 -- has been found to have a lower correlation with depression compared with other SOC instruments (e.g., the widely used 13-item of the SOC) [45].

Similar to Antonovsky’ s original tenet of SOC, we found that SOC was a stable trait in the present study. Many of the outcomes (except for those associated with pain, which was treated in the trial), changed very little over the 12 months. Therefore, we are unable to conclude that baseline SOC is not a predictor of change in HRQoL outcomes, mainly because SOC and these HRQoL outcomes remained stable over time. This lack of change over time was not surprising since the intervention did not specifically target these outcomes. According to Antonovsky [7,9], SOC remains stable throughout adulthood and is thereafter only minimally affected by traumatic life events. Several population-based studies supported this notion (i.e., SOC was found to be stable over time) [46-49]. However, more recent longitudinal studies derived from a more diverse group of non-pain related study participants and ranging from two to five years follow-up have found that SOC may vary over time [35,44,50,51]. To our knowledge, only one study has examined the extent to which SOC varied over time in patients with pain. In patients receiving laparoscopic cholecystectomy for gallstone disease, SOC was unstable over a 6 month period, changing more than 10% in 37% of the patients over 6 months [40]. Therefore, more longitudinal research is needed to examine the stability of SOC in different populations.

This study has several limitations. First, our sample was comprised entirely of US Veterans who received primary care from a single VAMC. Thus, our findings may be less generalizable to non-VA settings. However, in comparison to past studies with veterans, our sample included more women (18%) as well as higher educational attainment, employment rates and income. Second, there is the potential for selection bias because this study included only patients who were willing to enroll in a clinical trial. Third, the Cronbach’s α for the 3-item measure of SOC in the present study was .58, a value that is less than ideal. However, a systematic review of studies using the SOC-3 found that the Cronbach’s α ranged from .35 to .61 [46]. Schumann et al. [52] created a German version of SOC-3 and reported a Cronbach’s α of .45. Very brief scales typically have lower Cronbach’s α than longer scales. The fact that our principal analyses used the SOC as a binary measure based upon a validated cutpoint partly mitigates the moderate internal reliability of the 3-item SOC as a continuous measure. As Eriksson and Lindstrom [46] articulated, the intercorrelations between the SOC-3 and the original long SOC measures are satisfactory. The present study does not have validation data to compare the SOC-3 to the longer versions. Future research could explore the SOC-3 with longer versions of the SOC in English based samples. Fourth, our intervention focused on optimizing medications focused on pain. Future research should develop and implement an intervention that targets aspects of SOC and HRQoL to determine if SOC was a predictor of change in HRQoL.

Knowledge of the role of SOC in adapting to and coping with stress may equip health professionals for developing patient-centered care that incorporate SOC concepts to assist individuals suffering from musculoskeletal pain. Intervention strategies might be developed to help patients suffering from musculoskeletal pain; to identify their internal and external resources in order to strengthen their belief that their life is meaningful (i.e., the problems and demands of life are viewed as challenges instead of burdens), comprehensible (i.e., the circumstances that an individual will encounter in the future is viewed as predictable, ordered, and explicit), and manageable (i.e., the patient has the capacity at their disposal to deal with their pain). Thus, clinicians could help patients shift their focus away from crises, but instead view the encounters as balanced and meaningful [50].

## Conclusions

Based on a RCT involving a sample of 250 patients with persistent musculoskeletal pain who received care in VA primary care clinic, this study found that a strong SOC was independently associated with better HRQoL and self-efficacy, as well as less pain catastrophizing, after adjusting for sociodemographic and socioeconomic characteristics, and medical comorbidities. In contrast to most previous studies, the present study was able to adjust for presence of probable major depressive disorder. The significant associations between SOC and the aforementioned outcomes remained moderate in magnitude and statistically significant. The results also indicated that the “absence of major” depression had a strong and independent association with better HRQoL and pain-specific outcomes in all models. Finally, coping capability, like SOC, are needed to improve HRQoL for primary care patients suffering from musculoskeletal pain.

## Abbreviations

HRQoL: Health related quality of life; SOC: Sense of coherence; SCOPE: Stepped care to optimize pain care effectiveness; VA: Veterans affairs; ICD-9: International classification of diseases, ninth revision; BPI: Brief pain inventory; ASES: Arthritis self-efficacy scale; CSQ: Catastrophizing strategies questionnaire; SF-36: the 36-item short-form health survey; SF-12: the 12-item short-form health survey; PHQ-9: 9-item patient health questionnaire.

## Competing interests

The authors declare that they have no competing interest.

## Authors’ contributions

All authors contributed to and have approved the final manuscript. NRC contributed with the literature review, interpretation of the analysis, as well as drafting and revision of the manuscript. KK was responsible for the development and design of the study, interpretation of the analysis, diagnostic issues of the clinical sample and revision of the manuscript. SO was responsible to original outline of the manuscript. MJB contributed to the interpretation of the data and revision of the manuscript. EK contributed to the recruitment and diagnostic issues of the clinical sample and revision of the manuscript. ZY and JW contributed to both data management and data analysis.
